# Administration of galacto-oligosaccharide prebiotics in the Flinders Sensitive Line animal model of depression

**DOI:** 10.1136/bmjos-2018-000017

**Published:** 2019-05-25

**Authors:** Alexandra Bannach-Brown, Sandra Tillmann, Malcolm Robert MacLeod, Gregers Wegener

**Affiliations:** 1 Department of Clinical Medicine, Translational Neuropsychiatry Unit, Aarhus University, Aarhus, Denmark; 2 Centre for Research in Evidence-Based Medicine, Bond University, Gold Coast, Australia; 3 Centre for Clinical Brain Sciences, University of Edinburgh, Edinburgh, UK

**Keywords:** animal models of depression

## Abstract

**Introduction:**

Major depressive disorder is the leading source of disability globally and current pharmacological treatments are less than adequate. Animal models such as the Flinders Sensitive Line (FSL) rats are used to mimic aspects of the phenotype in the human disorder and to characterise candidate antidepressant agents. Communication between the gut microbiome and the brain may play an important role in psychiatric disorders such as depression. Interventions targeting the gut microbiota may serve as potential treatments for depression, and this drives increasing research into the effect of probiotics and prebiotics in neuropsychiatric disorders. Prebiotics, galacto-oligosaccharides and fructooligosaccharides that stimulate the activity of gut bacteria have been reported to have a positive impact, reducing anxiety and depressive-like phenotypes and stress-related physiology in mice and rats, as well as in humans. Bimuno, the commercially available beta-galacto-oligosaccharide, has been shown to increase gut microbiota diversity.

**Aim:**

Here, we aim to investigate the effect of Bimuno on rat anxiety-like and depressive-like behaviour and gut microbiota composition in the FSL model, a genetic model of depression, in comparison to their control, the Flinders Resistant Line (FRL) rats.

**Methods:**

Sixty-four male rats aged 5–7 weeks, 32 FSL and 32 FRL rats, will be randomised to receive Bimuno or control (4 g/kg) daily for 4 weeks. Animals will be tested by an experimenter unaware of group allocation on the forced swim test to assessed depressive-like behaviour, the elevated plus maze to assess anxiety-like behaviour and the open field test to assess locomotion. Animals will be weighed and food and water intake, per kilogram of bodyweight, will be recorded. Faeces will be collected from each animal prior to the start of the experiment and on the final day to assess the bacterial diversity and relative abundance of bacterial genera in the gut. All outcomes and statistical analysis will be carried out blinded to group allocation, group assignments will be revealed after raw data have been uploaded to Open Science Framework. Two-way analysis of variance will be carried out to investigate the effect of treatment (control or prebiotic) and strain (FSL or FRL) on depressive-like and anxiety-like behaviours.

## Introduction

Major depressive disorder (MDD) is the leading source of disability globally^1^ and treatment resistance among patients is roughly 50%.^2^ Therefore, better understanding mechanisms behind MDD and the search for potential effective and novel therapeutic targets are high research and healthcare priorities. Animal models are commonly used to mimic aspects of the phenotype of the human disorder and to characterise candidate antidepressant agents. The Flinders Sensitive Line (FSL) is a well-established and validated genetic model of depression.^3^ The FSL rats are bred to display cholinergic sensitivity and later found to display depressive-like behaviour in the forced swim test (FST), compared with their control strain, the Flinders Resistant Line (FRL) rats.^3^ FSL rats respond to acute and chronic antidepressant administration and display reduced hippocampal plasticity^4^ and elevated rapid eye movement (REM) sleep^5^ in comparison to FRL rats.

Communication between the gut microbiome and the brain may play a role in psychiatric disorders, with research focusing on the bidirectional signalling at the neural, hormonal and immunological levels.^6^ Interventions targeting the gut microbiota may serve as potential treatments for depression, and this drives increasing research into the effect of probiotics and prebiotics in neuropsychiatric disorders. Probiotics have been defined as ‘live organisms, that when ingested in adequate amounts, exert health benefits.’^7^ Several probiotic strains have been investigated in psychiatric disorders and have reported effects on behaviour and physiology in laboratory animals and humans (for a review see Wang and colleagues^8^). Commercially available probiotic products, ‘Ecological Barrier’ and ‘Probio’Stick’, have been tested in FSL rats.^9 10^ Prebiotics, defined as substrates that are selectively used by a host organism providing a health benefit,^11^ have also been reported to have a positive impact, reducing anxiety and depressive-like phenotypes and stress-related physiology in mice and rats^12–17^ and in humans.^18 19^ Further, prebiotics have been shown to increase the diversity of gut microbial composition, with evidence from mice^12^ and rats.^13^ Thompson and colleagues,^15^ however, showed no difference in gut microbiota composition in F344 rats receiving prebiotics. One prebiotic that is commercially available is Bimuno. Bimuno contains beta-galacto-oligosaccharide (B-GOS) produced from lactose in cow’s milk.^20^

These previous studies show promising effects of other prebiotics to reduce the depressive-like and anxiety-like phenotypes in stress models of depression,^12 14^ and resilience to a stressful exposure.^13 15^ Bimuno shows an effect on anxiety-like behaviour in response to a single lipopolysaccharide insult^17^ and GOS prebiotics have an effect on brain-derived neurotrophic factor (BDNF) levels.^16^ Based on previous studies showing promising effects of other prebiotics to reduce depressive-like and anxiety-like behaviours, we will investigate the effect of Bimuno on rat behaviour and gut microbiota composition in the FSL model, a genetic model of depression, in comparison to their control FRL rats.

This piece was initially submitted to *BMJ Open Science* as a registered report on 4 May 2018 and simultaneously uploaded to Open Science Framework (OSF). Registered reports are a publishing initiative designed to increase transparency and reproducibility of research by valuing high methodological quality. The registered reports framework involves a two-stage peer review process during the life-cycle of an article describing research; first, at the study design phase, and second at the stage of final publication of the findings.^21^ This initiative was introduced in major journals around 2013^22^ and was inspired by protocols for clinical trials. Registered reports in the context of in vivo animal experiments are a novel application of this publication framework. In comparison to animal experiments, clinical trials typically have a longer planning stage. Protocols for clinical trials are a requirement when seeking regulatory approval for interventions.^23^ Further, the sites of clinical trials are subject to inspections to check if the conduct of the trial is compliant with the protocol.^24^ Animal studies are often quicker to design and conduct with new hypotheses being generated quickly from previous studies, and the reporting guidelines for animal studies are less strictly adhered to. The protocols for animal studies that are conducted to support a Food and Drug Administration approval application require a sign-off from the sponsor.^25^

The increase of publishing registered reports sees many benefits, including an increase in measures to reduce the risk of bias and increased replicability.^26^ The application of the registered reports publishing framework to animal experiments can improve poor reporting, which threatens reproducibility.^27^ However, the framework may need amending to fully reap the benefits. This report was initially submitted on 4 May 2018. The first peer review comments were received on 11 September 2018 and the second round of peer review comments was received in November, 6 months later. Within the time constraints of a 3-year funding window of a PhD project, the experiment was conducted without completing the peer review process at the study design phase. The piece is therefore to be submitted as a ‘Protocol’.

### Hypotheses

We hypothesise that FSL animals receiving Bimuno prebiotics will display reduced depressive-like behaviour in the FST and reduced anxiety-like behaviour in the elevated plus maze (EPM) in comparison to control (substance without active ingredients, Bimuno free sugars [BFS]). We aim to contribute to the literature describing the behavioural effects of prebiotics in animal models of depression. As our secondary outcome, we hypothesise that FSL animals receiving prebiotics will display increased diversity in the gut microbiome, in comparison to FSL animals receiving control, as measured on true beta diversity. We want to analyse gut microbiome diversity because we hypothesise that this is the mechanism through which prebiotics influence behaviour, we therefore aim to shed light on the commensal influence of prebiotics.^28^ We hypothesise that animals receiving Bimuno prebiotics will have altered weight and food intake in comparison to animals receiving control.

## Methods

### Animals

Male FSL and FRL rats aged 5–7 weeks bred in-house at Translational Neuropsychiatry Unit, Aarhus University, will be used. Animals are bred in a closed breeding colony. Animals will be transferred from the closed breeding colony to a conventional animal facility and housed here for the duration of the experiment. All animals bred in-house are checked every 3 months for infectious agents in accordance with the Federation of European Laboratory Animal Science Associations (FELASA) recommendations.^29^ Animals did not have any known infections at the start of the experiment and were healthy, as assessed by FELASA-accredited in-house animal technicians.

Animals will be housed in pairs in standard cages with a plastic bottom and metal rack top half, purchased from Tecniplast, Italy (Cage 1291H Eurostandard Type III H, 425×266×185 mm). The bedding material in each cage will be made out of wooden chips (aspen wood from Tapvei, Finland) along with access to a tunnel shelter, nesting material and a wooden stick. Animals will be maintained in a 12-hour light/dark cycle with lights off at 13:00 hours. Seven days prior to the start of the experiment, the animals will be moved to the animal housing facility and the new lightning regime will start immediately. Animals will be under the care of FELASA-accredited in-house animal technicians. Animals will have tap water and standard chow (purchased from Brogaarden, Altomen 1324).

### Power calculation to determine the number of animals

Our sample size calculations are based on published behavioural findings from Burokas and colleagues^12^ and McVey Neufeld and colleagues.^14^

Data were extracted from Burokas *et al*,^12^ who investigated the effects of prebiotics in the FST using male C57L/6J mice (figure 6D in the publication). These data (mean, SEM and group numbers) were used to run a one-way analysis of variance (ANOVA) and determine an eta squared (=SSbetween/SStotal) of 0.579. This eta squared value was used to compute the effect size, f= $\left(\sqrt{\left(\frac{eta^{2}}{1-eta^{2}}\right)}\right)$ , which is 1.1747. This effect size was used in the power calculation carried out in R (V.3.4.3) using the function ‘pwr.anova.test’. A significance level of 0.01 and a power of 0.9 were chosen. This gave the result of six experimental units per group. An experimental unit is the entity subjected to an intervention independently of all other units where it is possible to assign two experimental units to different treatment groups.^30^

**Figure 1:**
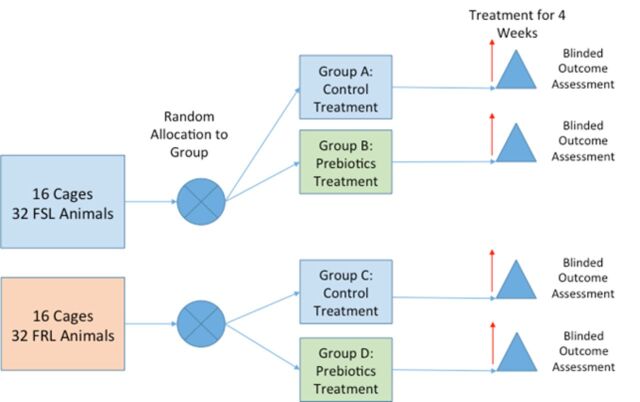
Experimental design set-up. FRL, Flinders Resistant Line; FSL, Flinders Sensitive Line.

Data were extracted from McVey Neufeld *et al*,^14^ who used prebiotics and probiotics in a maternal separation model of depression in the open field using male Sprague-Dawley rats. Data are from the amount of time spent in the centre of the open field (figure 1B in the publication) for the model group. These data (mean, SD or SEM, and group numbers) were used to run a one-way ANOVA and determine an eta squared (=SSbetween/SStotal) of 0.522. This eta squared value was used to compute effect size, f= $\left(\sqrt{\left(\frac{eta^{2}}{1-eta^{2}}\right)}\right)$ , which is 1.046. This effect size was used in the power calculation carried out in R (V.3.4.3) using the function ‘pwr.anova.test’. A significance level of 0.01 and a power of 0.9 were chosen. This gave the result of six experimental units per group.

Based on the a priori sample size calculations above and experience from previous in-house experiments, a conservative estimate of sample size for this study of eight experimental units per group was selected. This number is two per group larger than the power calculation and was selected to account for possible attrition or possible exclusions throughout the experiment (see criteria below). With eight experimental units per group, power of 90% and a significance level of 0.01, we are powered to detect an effect of f=0.86. This effect size we consider biologically relevant, in order to see a relevant reduction in immobility behaviour in the FST. The full R code for these calculations is provided on the Open Science Framework project:.

### Prebiotics administration

The prebiotic and control treatment will be administered for 28 consecutive days (4 weeks). The treatments will be administered within the first hour after lights off, the first hour of the animals’ active phase.

We will use the commercially available prebiotic product ‘Bimuno’ Powder (Bimuno, UK), which contains B-GOS. A dose of 4 g/kg dissolved in tap water will be used per animal per day, administered by syringe feeding. The dose will be adjusted each week according to the weight of the animals. This prebiotic was chosen due to its superior effect over fructooligosaccharides.^16^ This dose was given to recreate the findings in previous literature.^16 31^

### Control administration

The control for the prebiotics will be the BFS (consisting of 50% lactose, 27% glucose and 23% galactose). The control will be administered at a dose of 4 g/kg/day. This follows the dosing regimen of previous literature.^16 31^ This control will be administered simultaneously to the prebiotics administration via syringe feeding in a volume of 2 mL. The dose will be adjusted each week according to the weight of the animals.

### Syringe feeding details

Treatment will be administered via syringe feeding; the prebiotic, within a sweetened vehicle of glucose, is mixed with tap water, and added to a syringe. This is a newly established method for the accurate individual dosing of probiotics in rats.^32^ With a training phase of roughly 3–4 days, to allow the rats to become accustomed to the administration and the taste, the rats willingly consume the mixture and approach the edge of the cage when the syringe is presented. This new method has been used for volumes of probiotic+vehicle solution up to 3 mL. This method of administration has been chosen to reduce the stress associated with oral gavage, and to increase the accuracy of dosing with administration of prebiotics in drinking water. In this experiment, the prebiotic Bimuno will be added to tap water to give a total volume of 2 mL, as the smaller the volume, the sweeter the solution, which is thought to be more desirable for the rats to consume. Animals will be fed at the start of the active cycle, within the first hour after lights off.

### Measures to reduce the risk of bias

#### Randomisation to treatment and control and allocation concealment

On the first day of the experiment, animals will be moved from the breeding facility into the experimental facility. Animals are pair housed; the two animals in each cage will be the same strain and will receive the same treatment. Cages will be randomly assigned to a group, treatment or control, to ensure allocation concealment during the handling and administration of treatment throughout the experiment. Randomisation will be carried out using block randomisation with the online tool, the Sealed Envelope (https://www.sealedenvelope.com/simple-randomiser/v1/lists), by a colleague not involved in the day-to-day running of the experiment. Cages will be labelled with a unique randomisation code (eg, GU9, LI3, and so on) and a list of which treatments are given to which cage will be read off each day. This is to minimise potential unconscious bias by ‘remembering’ which cages get which treatment. Treatments will be identified as A or B. The cages (the experimental unit) will be assigned randomly to treatment and the observational unit is the individual animal where the outcome of interest is measured. The observational unit (the animal) is nested within the experimental unit. The order of the cages will be randomised in the racks at the beginning of the experiment so as to reduce possible effects from air-conditioning vents and/or being closer to the door. The placement of the cage will not be taken into consideration as a variable during analysis of the outcome data. Animals will have 7 days to acclimatise to new housing facilities. When the experiment and treatment administration begins, the experimenter will be blinded to which solution (prebiotic or vehicle control) each rat receives. The prebiotics and free sugars are delivered in unmarked sachets (only with company logo and batch number) of 3.56 g. Sachets will be removed from their identifying boxes and moved into plastic boxes marked, for example, A+B, to signify which groups will receive the sachets, by a colleague not involved in the day-to-day running of the experiment. They will put enough sachets for the duration of the experiment. This allows the primary experimenter to prepare and administer the treatments each day for 28 days in a blinded manner.

#### Blinded assessment of primary outcome

The primary outcome is the FST. This outcome is recorded on video and scored manually. The videos will be assessed blinded, before the group identity of the animals is revealed. The same procedure will be carried out for the open field test. All videos will be analysed after all behavioural outcomes have been carried out. The primary experimenter will be formally unblinded to the true group identity after data analysis files have been uploaded to OSF.

### Outcome assessment

Behavioural assessment will occur during the rats’ active phase, starting approximately 1 hour after administration of prebiotics, 1 hour after lights off, and lasting approximately 3 hours, until 4 hours after lights off.

#### Forced swim test

On day 26 of the experiment, 1 hour after lights off, at the start of the animals’ active phase, the FST will be performed. Clear glass cylinders (60 cm h×24 cm Ø) filled with water up to 40 cm will be used. The temperature of the water will be kept at 25°C±1°C. On the first day, the preswim session, the animals will be placed in the tanks for 15 min. On the second day of testing, animals will be placed into the tanks for 5 min. Both sessions will be recorded by video camera. Both testing sessions will be conducted in red light conditions. Three behavioural parameters will be assessed from the video footage: passive behaviour, immobility, and two active behaviours, swimming and climbing behaviours. Passive behaviour is defined as ‘the rat making no further movements beyond those needed to keep its head above the water.’^9^ For each 5 s period, the predominant behaviour will be recorded (immobility, swimming or climbing). Counts of behaviour on the three behaviours will be summed and time spent across the swim session (5 min) will be calculated, for example, 12×5 s periods of immobility=60 s out of 5 min, 27×5 s periods of swimming=135 s out of 5 min, 21×5 s periods of climbing=105 s out of 5 min. All swimming sessions will be scored by an experimenter blinded to the group assignment of the animals.

#### Open field test

Locomotor activity will be assessed on day 27, immediately prior to the second FST session. Locomotor activity will be assessed in a 100 cm×100 cm (×20 cm h) black open field arena. Each animal will be placed in the arena in the same starting location. The animals will be assessed for 15 min in red light because they will be tested in their active phase at 1 hour after lights off. All sessions will be video recorded and analysed using Noldus EthoVision XT9. Locomotor activity will be measured from the video recording, assessed as the distance each animal moved in centimetres. The arena will be cleaned with ethanol between each animal. All video recordings will be scored by an experimenter blinded to the group assignment of the animals.

#### Elevated plus maze

Anxiety behaviour will be assessed on day 24 in the EPM. The plus-shaped maze has two open arms and two closed arms (length: 50 cm×width: 10 cm) and the centre zone measures 10 cm×10 cm. Each animal will be placed in the arena in the centre, facing the same open arm. Animals will be assessed for 5 min and will be tested during their active phase, 1 hour after lights off. The light intensity in the open arms will be 80–100 lx and 20 lx in the closed arms. Animals will be kept in an adjacent dark experimental room and moved individually into the bright experimental room for testing. All sessions will be video recorded and analysed using Noldus EthoVision XT9. Anxiety behaviour will be measured by calculating the time spent in the open arms in proportion to the time spent in the open arms and closed arms and number of entries into open arms (defined as the entire body of the rat in the open arm). The arena will be cleaned with ethanol between each animal. All video recordings will be scored by an experimenter blinded to the group assignment of the animals.

#### Body weight and food consumption

Animals will be weighed every week throughout the experiment to assess if prebiotics administration influences weight gain, and also to adjust the dose of the prebiotics or control administered (4 g/kg). Weekly food and water intake (per kg body weight) in the home cage will also be recorded.

#### Microbiota analysis

Faecal boli will be collected at the start of the experiment (day 1) and on day of euthanisation (day 28). Faecal boli at the start and end of the experiment will be collected directly from each animal into sterile tubes and frozen and stored at −80°C. Faecal boli will be used to analyse the composition of gut microbiota. DNA will be isolated from the faecal boli using the isopropanol DNA extraction method.^33^ 16S rRNA amplicon sequencing on V4-5 will be carried out on faecal boli from individual animals. Broad sequencing will be carried out by an external biotech company, DNA Sense, in Aalborg, Denmark. The alpha diversity, the beta diversity and the abundance of genera, more specifically, the relative abundance of *Bifidobacterium* and *Lactobacillus* genera, will be analysed as GOS prebiotics have previously been shown to enhance diversity and these specific genera.^12 34 35^

#### Dissection

Animals will be euthanised on day 28 and the whole brain will be removed. Brains will be stored in formaldehyde at −20°C. Brain chemistry analyses to assess neurotransmitter receptor mRNA expression and growth factors, such as BDNF, will be carried out, funding permitting. A protocol annex for these additional analyses will be submitted to the OSF prior to commencement.

### Exclusion criteria

1. Animals will be excluded from the study if they, at any point during the study, display illness as assessed by trained, in-house veterinarians on a daily basis. Animals will be euthanised immediately to end suffering.
2. No animals will be excluded from the statistical analysis if they successfully complete all aspects of the study. Data for each animal will be included in analysis of the individual tests if they completed the test. If an animal does not complete the open field test they will be removed from the FST analysis as we would be unable to exclude the impact of hyperlocomotion of activity in the FST.
3. Potential reasons why an animal might not complete an aspect of the study include technical difficulties during the video recording, or other circumstances where the raw data or data collection process has been compromised and statistical analysis is not possible.
4. There are no exclusion criteria based on performance on behavioural tests.
5. Potential reasons why an animal might be excluded from microbial analysis include failure to produce faecal boli on two separate occasions during the day of collection, or technical difficulties during the sequencing.

### Experimental procedure

Step 1: Animals are bred in-house. At 5–7 weeks of age, rats will be moved into experimental housing and have a 7-day acclimatisation/habitation period to the animal housing facility prior to the start of the experiment. The FSL and FRL rats will be randomised into two groups: treatment and control.

Step 2: On day 1 of the experiment, each rat will be weighed and faecal boli will be collected. Then the first administration of prebiotics or control will be given.

Step 3: Animals will remain continuously on this treatment regimen for 4 weeks (28 days). Rats will be weighed every week. The animals’ daily food and water consumption will be recorded.

Step 4: On day 24, animals will be subjected to the EPM.

Step 5: On day 26, animals will be subjected to the preswim of the FST.

Step 6: On day 27, the open field test and the FST will be carried out.

Step 7: On day 28, animals will be euthanised and brains will be harvested from all animals.

The timing of outcome assessments is displayed in table 1and Figure 1.

**Table 1:** Timing of outcome measure administration to each group

Model	Treatment	Length of administration (weeks)	Elevated plus maze	FST and open field	Euthanasia
FSL	Prebiotics	4	Day 24	Day 27	Day 28
FSL	Control	4	Day 24	Day 27	Day 28
FRL	Prebiotics	4	Day 24	Day 27	Day 28
FRL	Control	4	Day 24	Day 27	Day 28

FRL, Flinders Resistant Line; FSL, Flinders Sensitive Line; FST, forced swim test.

### Data analysis pipeline

Data from the open field and FST will be recorded in EthoVision. Videos will be stored on a network drive. Video from each animal will be scored blinded to the animal’s group assignment. Potential data transformation and all statistical analysis will be carried out in R Studio using the latest version of R.

### Statistical analysis

First, the data from individual animals will be averaged per cage to obtain a value for the experimental unit. A Student’s t-test will be performed to compare the performance in the FST between the FSL and FRL rats, to confirm that the FSL rats do indeed display increased depressive-like behaviour. To test the hypothesis that probiotics improve depressive-like behaviour on the FST and anxiety-like behaviour in the EPM, the primary outcome being immobility time, a two-way ANOVA will be conducted with two independent variables: treatment (control or prebiotic) and strain (FSL or FRL). Group differences will be investigated with a Tukey post hoc test. If data are not normally distributed, a base-10 log transformation will be carried out. If data do not meet assumptions for homogeneity of variances in the ANOVA or t-test, a Welch correction will be used. For the ANOVA, a Kruskal-Wallis test will be conducted if residuals are not normally distributed. A Tukey post hoc test will be carried out to investigate group differences; this will be carried out also if data are corrected/transformed data. All raw data, transformed (if applicable), and data analysis code will be uploaded to Open Science Framework.

Principal coordinate analysis will be conducted based on 16s rRNA sequencing data to quantify the beta diversity, clustering based on operational taxonomic unit, and to measure the relative abundance of genera. The relative abundance of *Bifidobacterium* and *Lactobacillus* genera, as well as total bacteria, will be compared between groups.

## Discussion, limitations and external validity considerations

The sample size calculation used to estimate the number of animals to be used is based on one study in mice using the FST^12^ and one study in rats using the open field test.^14^ There are limitations to the use of the outcomes from these papers. First, we do not have published data from the effects of prebiotics in rats for our primary outcome, the FST. Therefore, we combined the use of published FST data from mice and published data from rats in the open field, our secondary outcome. This may yield an inaccurate appropriate sample size calculation; however, we are using the available published data to provide a best estimate.

We did not control for littermate effects in this study. Littermate effects could impact the similarity of microbiota between and within cages. If animals in a cage were from the same litter, they have more similar microbiota compared with animals in a cage from separate litters.^36^ If animals in a treatment group are from two to three litters compared with animals from more than three or four litters, this is hypothesised to be of higher variability. We have aimed to stabilise the effects and reduce cage variability somewhat by cohousing the animals for 7 days prior to the start of the experiment. However, the effect of littermate effects on microbiota variability is unknown in this experiment.

We aimed to measure the starting microbiome of each animal and measure the impact of prebiotics on the gut microbiome by taking faecal samples at the beginning and end of the experiment. Rather than controlling for similar gut microbiota between the animals at the start of the experiment, which would reduce the variability observed, also does not reflect a naturalistic environment.

The choice of model in this experiment will be the FSL rats. This model of depression has a moderate level of face validity^3^ sharing some similarities with the human disorder, such as elevated REM sleep and increased passive behaviour following stress.^3^ However, this model does not reflect the full heterogeneity of symptoms observed in depression in humans, and may not reflect the complex interplay between genetics and environment that contribute to the development of depression in humans. Therefore, there may be limited translatability of the findings from this study directly to human populations.

This experiment will be conducted in male animals only. To ensure findings are externally valid in both sexes, a follow-up experiment will need to be conducted including both sexes to analyse the sex difference in the effects of prebiotics.

The choices of behavioural outcome measures that will be used in the experiment are EPM, open field test and the FST. The limitations of these behavioural tests have been extensively discussed elsewhere^37^ (Spruijt *et al*, 2014^38^), we acknowledge the issues with lack of reproducibility across studies. The order of administration of the tests was chosen to be conducted from least to most stress inducing. All animals will be tested on behavioural assessments in the same order: EPM, 48 hours’ rest, preswim of the FST, 24 hours’ rest, open field test of locomotion and FST test session directly after.

The prebiotics will be mixed with tap water. This method of administration is similar to how the prebiotics would be administered in human studies. Although there has not been an investigation into the effects of tap water versus autoclaved water, we thought this method of administration would maintain ecological validity and mimic the method of administration in human consumption of the prebiotics.

Due to time and resource constraints, this report was not submitted as a registered report and instead submitted as a protocol. Similar projects conducting animal behaviour experiments in the laboratory will likely be subject to similar time pressures and therefore a peer review process of longer than 6 months is not feasible for effective implementation of registered reports in animal experiments. As the registered reports publishing framework is being trialled for animal experiments, we can consider the merits and limitations of this approach. How can the benefits of registered reports be reaped while fitting into the current academic and scientific model used in animal experiments? The aim is to ensure the benefits of openness and transparency to improve quality of both the reporting and the conduct of animal experiments, and to ensure the results from experiments are as accurate as possible where hypotheses are supported with adequately powered studies to reduce the risk of false positive and false negative findings.

Perhaps the current peer review process is not suitable for implementing registered reports for animal experiments. The current model in animal experimental work involves the conduct of many small experiments. Hypotheses change rapidly, and many exploratory and confirmatory experiments are conducted. For registered reports to be useful in the context of animal experiments, peer review needs to be incredibly rapid in order to allow for the fast life-cycle of this research. An alternative review process may be required to expedite peer review comments. One potential is an online forum or portal where authors and reviewers can post publications and comments, and discuss. Another alternative may be simply to promote the more widespread use of protocol registration on a protocol platform. Platforms such as OSF can host the protocols and associated materials. Preregistration of animal experiments has been trialled by preclinicaltrials.eu. Protocol registration platforms can reap most of the benefits of registered reports; stating whether an experiment is exploratory or confirmatory, having a clear predefined hypothesis where confirmatory and stating the intended methodology and statistical analysis plan all contribute to transparent work and reduce research waste in unnecessary replication of experiments. Reporting a priori sample size calculation can also contribute to improving the quality of confirmatory experiments performed.

Another alternative may be to rethink and restructure short research projects. Current metrics of success in academia are based on the number of successful or ‘significant’ findings from experiments, and novelty of hypotheses or methodology is highly rewarded. A restructure would value and incentivise projects that ensure high-quality experiments are conducted, findings are replicated and have external validity. It may be necessary to develop new metrics for measuring contribution to projects. One example of implementing this idea in practice comes from Button and Munafo in psychology,^39^ setting up a consortium across universities for student projects. Best practices and protocols for projects are established collectively and data on the same research question are collected at multiple sites. An alternative incentive system is implemented by using the inclusive authorship conventions commonly used in genetics and physics. In principle, it is easy to set up such a consortium that collaborates on testing hypotheses in animal experiments. Testing hypotheses across animal labs would ensure that findings are robust to variability in lab settings. By shifting the framework to a collaborative approach and rewarding contribution to the collaboration, such as is used in these consortia, the benefits of conducting high-quality research can be reaped. Several possible changes can be made to the current paradigm to ensure the benefits of registered reports can be reaped for animal experiments. These are not mutually exclusive and may be implemented together for added benefit.

## References

[1] World Health Organization (WHO). Depression. “A Global Public Health Concern Developed” Authors: Marina Marcus, M. Taghi Yasamy, Mark van Ommeren, and Dan Chisholm, Shekhar Saxena: WHO Department of Mental Health and Substance Abuse, 2012.

[2] Thomas L , Kessler D , Campbell J , et al . Prevalence of treatment-resistant depression in primary care: cross-sectional data. Br J Gen Pract 2013;63:e852–8. 10.3399/bjgp13X675430 24351501 PMC3839394

[3] Overstreet DH , Wegener G . The flinders sensitive line rat model of depression—25 years and still producing. Pharmacol Rev 2013;65:143–55. 10.1124/pr.111.005397 23319547

[4] Chen F , Madsen TM , Wegener G , et al . Imipramine treatment increases the number of hippocampal synapses and neurons in a genetic animal model of depression. Hippocampus 2010;20:1376–84. 10.1002/hipo.20718 19921703

[5] Benca RM , Overstreet DE , Gilliland MA , et al . Increased basal REM sleep but no difference in dark induction or light suppression of REM sleep in flinders rats with cholinergic supersensitivity. Neuropsychopharmacology 1996;15:45–51. 10.1016/0893-133X(95)00154-6 8797191

[6] Cryan JF , O’Mahony SM . The microbiome-gut-brain axis: from bowel to behavior. Neurogastroenterol Motil 2011;23:187–92. 10.1111/j.1365-2982.2010.01664.x 21303428

[7] Dinan TG , Stanton C , Cryan JF . Psychobiotics: a novel class of psychotropic. Biol Psychiatry 2013 74:720–6. 10.1016/j.biopsych.2013.05.001 23759244

[8] Wang H , Lee IS , Braun C , et al . Effect of probiotics on central nervous system functions in animals and humans: a systematic review. J Neurogastroenterol Motil 2016;22:589–605. 10.5056/jnm16018 27413138 PMC5056568

[9] Abildgaard A , Elfving B , Hokland M , et al . Probiotic treatment protects against the pro-depressant-like effect of high-fat diet in Flinders Sensitive Line rats. Brain Behav Immun 2017;65:33–42. 10.1016/j.bbi.2017.04.017 28450222

[10] Tillmann S , Awwad HM , Eskelund AR , et al . Probiotics affect one-carbon metabolites and catecholamines in a genetic rat model of depression. Mol Nutr Food Res 2018;62:e1701070. 10.1002/mnfr.201701070 29453804 PMC5900923

[11] Gibson GR , Hutkins R , Sanders ME , et al . Expert consensus document: The International Scientific Association for Probiotics and Prebiotics (ISAPP) consensus statement on the definition and scope of prebiotics. Nat Rev Gastroenterol Hepatol 2017;14:491. 10.1038/nrgastro.2017.75 28611480

[12] Burokas A , Arboleya S , Moloney RD , et al . Targeting the microbiota-gut-brain axis: prebiotics have anxiolytic and antidepressant-like effects and reverse the impact of chronic stress in Mice. Biol Psychiatry 2017;82:472–87. 10.1016/j.biopsych.2016.12.031 28242013

[13] Mika A , Day HE , Martinez A , et al . Early life diets with prebiotics and bioactive milk fractions attenuate the impact of stress on learned helplessness behaviours and alter gene expression within neural circuits important for stress resistance. Eur J Neurosci 2017;45:342–57. 10.1111/ejn.13444 27763700

[14] McVey Neufeld KA , O’Mahony SM , Hoban AE , et al . Neurobehavioural effects of Lactobacillus rhamnosus GG alone and in combination with prebiotics polydextrose and galactooligosaccharide in male rats exposed to early-life stress. Nutr Neurosci 2017:1–10. 10.1080/1028415X.2017.1397875 29173065

[15] Thompson RS , Roller R , Mika A , et al . Dietary prebiotics and bioactive milk fractions improve nrem sleep, enhance rem sleep rebound and attenuate the stress-induced decrease in diurnal temperature and gut microbial alpha diversity. Front Behav Neurosci 2016;10:240. 10.3389/fnbeh.2016.00240 28119579 PMC5223485

[16] Savignac HM , Corona G , Mills H , et al . Prebiotic feeding elevates central brain derived neurotrophic factor, N-methyl-D-aspartate receptor subunits and D-serine. Neurochem Int 2013;63:756–64. 10.1016/j.neuint.2013.10.006 24140431 PMC3858812

[17] Savignac HM , Couch Y , Stratford M , et al . Prebiotic administration normalizes lipopolysaccharide (LPS)-induced anxiety and cortical 5-HT2A receptor and IL1-β levels in male mice. Brain Behav Immun 2016;52:120–31. 10.1016/j.bbi.2015.10.007 26476141 PMC4927692

[18] Schmidt K , Cowen PJ , Harmer CJ , et al . Prebiotic intake reduces the waking cortisol response and alters emotional bias in healthy volunteers. Psychopharmacology 2015;232:1793–801. 10.1007/s00213-014-3810-0 25449699 PMC4410136

[19] Kazemi A , Noorbala AA , Azam K , et al . Effect of probiotic and prebiotic vs placebo on psychological outcomes in patients with major depressive disorder: A randomized clinical trial. Clin Nutr 2019;38:522–8. 10.1016/j.clnu.2018.04.010 29731182

[20] Bimuno®. United Kindgom. 2018 https://www.bimuno.com/ (Accessed 20th Apr 2018).

[21] Centre for Open Science. Registered Reports. 2018 https://cos.io/rr/ (Accessed 13 Dec 2018).

[22] Chambers C . Registered Reports: A change in scientific publishing”. 2014 https://www.elsevier.com/reviewers-update/story/innovation-in-publishing/registered-reports-a-step-change-in-scientific-publishing (Accessed 13 Dec 2018).

[23] ICH Expert Working Group. ICH harmonised tripartite guideline: guideline for good clinical practice E6(R1) dated 10 June 1996 including post step 4 corrections. Guildford: Canary, 1996.

[24] Food & Drug Administration, U.S. Department of Health and Human Services. “Information Sheet Guidance For IRBs, Clinical Investigators, and Sponsors: FDA Inspections of Clinical Investigators”. 2010 https://www.fda.gov/downloads/RegulatoryInformation/Guidances/UCM126553.pdf (Accessed 13 Dec 2018).

[25] Food & Drug Administration, U.S. Department of Health and Human Services. 21 CFR Part 58.1: Good Laboratory Practice for Nonclinical Laboratory Studies”. 2018 https://www.accessdata.fda.gov/scripts/cdrh/cfdocs/cfcfr/CFRSearch.cfm?fr=58.120 (Accessed 13 Dec 2018).

[26] Odutayo A , Emdin CA , Hsiao AJ , et al . Association between trial registration and positive study findings: cross sectional study (Epidemiological Study of Randomized Trials-ESORT). BMJ 2017;356:j917. 10.1136/bmj.j917 28292744 PMC6283391

[27] Freedman LP , Cockburn IM , Simcoe TS . The Economics of Reproducibility in Preclinical Research. PLoS Biol 2015;13:e1002165. 10.1371/journal.pbio.1002165 26057340 PMC4461318

[28] Sherwin E , Sandhu KV , Dinan TG , et al . May the force be with you: the light and dark sides of the microbiota-gut-brain axis in neuropsychiatry. CNS Drugs 2016;30:1019–41. 10.1007/s40263-016-0370-3 27417321 PMC5078156

[29] Mähler Convenor M , Berard M , Feinstein R , et al . FELASA recommendations for the health monitoring of mouse, rat, hamster, guinea pig and rabbit colonies in breeding and experimental units. Lab Anim 2014;48:178–92. 10.1177/0023677213516312 24496575

[30] National Centre for the Replacement, Refinement and Reduction of Animals in Research (NC3Rs). Experimental Unit. 2018 https://eda.nc3rs.org.uk/experimental-design-unit (Accessed 03 May 2018).

[31] Williams NC , Johnson MA , Shaw DE , et al . A prebiotic galactooligosaccharide mixture reduces severity of hyperpnoea-induced bronchoconstriction and markers of airway inflammation. Br J Nutr 2016;116:798–804. 10.1017/S0007114516002762 27523186

[32] Tillmann S , Wegener G . Syringe-feeding as a novel delivery method for accurate individual dosing of probiotics in rats. Benef Microbes 2018;9:311–5. 10.3920/BM2017.0127 29264968

[33] Hart ML , Meyer A , Johnson PJ , et al . Comparative evaluation of dna extraction methods from feces of multiple host species for downstream next-generation sequencing. PLoS One 2015;10:e0143334. 10.1371/journal.pone.0143334 26599606 PMC4657925

[34] Monteagudo-Mera A , Arthur JC , Jobin C , et al . High purity galacto-oligosaccharides enhance specific *Bifidobacterium* species and their metabolic activity in the mouse gut microbiome. Benef Microbes 2016;7:247–64. 10.3920/BM2015.0114 26839072 PMC4974821

[35] Liu F , Li P , Chen M , et al . Fructooligosaccharide (FOS) and Galactooligosaccharide (GOS) Increase *Bifidobacterium* but reduce butyrate producing bacteria with adverse glycemic metabolism in healthy young population. Sci Rep 2017;7:11789. 10.1038/s41598-017-10722-2 28924143 PMC5603605

[36] Moore RJ , Stanley D . Experimental design considerations in microbiota/inflammation studies. Clin Transl Immunology 2016;5:e92. 10.1038/cti.2016.41 27525065 PMC4973323

[37] Commons KG , Cholanians AB , Babb JA , et al . The rodent forced swim test measures stress-coping strategy, not depression-like behavior. ACS Chem Neurosci 2017;8:955–60. 10.1021/acschemneuro.7b00042 28287253 PMC5518600

[38] Spruijt BM , Peters SM , de Heer RC , et al . Reproducibility and relevance of future behavioral sciences should benefit from a cross fertilization of past recommendations and today’s technology: "Back to the future". J Neurosci Methods 2014;234:2–12. 10.1016/j.jneumeth.2014.03.001 24632384

[39] Button KS , Lawrence NS , Chambers CD , et al . Instilling scientific rigour at the grassroots. Psychologist 2016;29:158–9.

